# Science battles viral diseases: a roundtable discussion with leading experts on COVID-19, hepatitis and AIDS

**DOI:** 10.1093/nsr/nwac277

**Published:** 2022-12-06

**Authors:** He Zhu

**Affiliations:** Science and news editor at the editorial office of NSR in Beijing, China

## Abstract

The Science Popularization and Education Committee of the Academic Divisions of the Chinese Academy of Sciences (CASAD) invited the CAS Institutes of Science and Development (CASISD) and National Science Review (NSR) to organize a roundtable discussion of viral infectious diseases on 11 October 2022. During this extensive discussion, experts introduced the history and background of virus and viral infectious diseases. They explained the function of human's immune system. In addition, they answered frequently asked questions by the public such as the efficacy and safety of COVID vaccines, the cost of disease treatment and the threat of latest monkey pox outbreak.

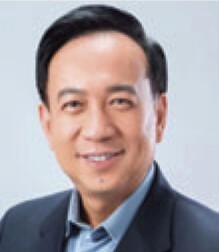

**Chen Dong** (董晨)

Professor, director of Shanghai Immune Therapy Institute

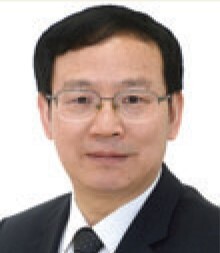

**Fu-Sheng Wang** (王福生)

MD., director of the Department of Infectious Diseases, Fifth Medical Center of Chinese PLA General Hospital, National Clinical Research Center for Infectious Diseases

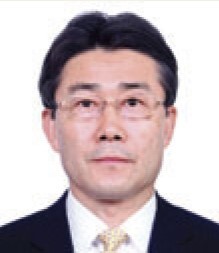

**George Fu Gao** (高福)

Professor, former director-general of Chinese Center for Disease Control and Prevention (China CDC)

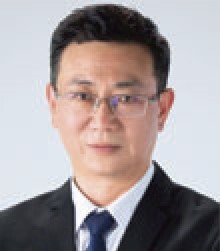

**Zhonghe Zhou** (周忠和, Chair)

Associate director of the Science Popularization and Education Committee of CASAD, professor of CAS Institute of Vertebrate Paleontology and Paleoanthropology and associate editor-in-chief of NSR

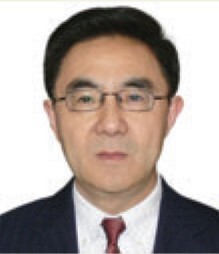

**Yiming Shao** (邵一鸣)

Professor, deputy director of State Key Laboratory for Infectious Disease Control and Prevention, former chief expert on AIDS, China CDC

**Zhou:** Today's discussion will be focused on virus, immunity and vaccine. We will answer hot topic questions of the public. The first questions are regarding basic aspects of viruses. What viruses cause infectious diseases and why we should pay attention to them? Prof. Gao, would you like to start?

**Gao:** Before the onset of COVID-19, the general public may not have paid a lot of attention to viruses. Now it is the talk of the entire world. Like a person, a virus is a living organism. It appeared on earth before humans; its evolution greatly preceded our evolution; its coexistence with us had affected our lives many times before. Fortunately, some terrible viruses such as smallpox and polio are mostly eradicated or eliminated, but some mild viruses are part of our daily lives.

**Zhou:** Thank you. Dr. Wang, would you like to introduce the history of humans’ interaction with viruses?

**Wang:** I agree with Prof. Gao. Humans have always coexisted with microbes such as viruses and bacteria. Some viruses can cause emerging infectious diseases and severe infectious diseases. The history of the past few centuries shows us that viral infectious diseases have been particularly detrimental to human lives. With modern medicine, infectious diseases caused by bacteria can be controlled and treated very well. But we still lack effective therapeutic drugs and treatment options for some emerging infectious diseases and severe infectious diseases caused by infectious viruses and we cannot adequately control them. In recent years, viruses have frequently caused pandemics or epidemics.

(viruses’) coexistence with us had affected our lives many times before.

—Fu Gao

**Zhou:** I see. Infectious diseases can be caused by viruses or bacteria. Are viral infectious diseases more serious?

**Shao:** Yes, you are correct. According to national statistics of fatalities caused by all infectious diseases in China, the top five combined account for 99.7% of all fatalities, the most deadly is AIDS and number three, four and five are all viral diseases, except tuberculosis as number two. Globally, a notable incident was the influenza pandemic of 1918 that claimed more than 50 million lives, several times the death toll of World War I. Virus is truly the worst threat to human lives. In the first two decades of this century so far, we have already had three corona virus outbreaks: SARS in 2003, MERS in 2014 and COVID-19 in 2019.

**Zhou:** So viruses have been a dangerous threat for a long time. My next question for Prof. Dong is: have viruses evolved to be more dangerous than before?

**Dong:** This is an interesting question. In the course of human evolution, the primary defense against viruses is our own immune system. Only in the past one hundred years have vaccines started to play a crucial role. In a modern society, sanitation has been greatly improved in terms of the surfaces we touch and food we eat. One remaining concern is air quality and respiratory health. So our living environment is constantly changing and viruses are constantly adapting to us. In turn, we are adapting to the viruses around us.

**Zhou:** Earlier we talked about a few viruses that have been eliminated or eradicated. But we have to coexist with most others, Is that correct, Prof. Dong?

**Dong:** Yes, a good example is the influenza virus. In addition, the common cold contains several types of corona virus. Many corona viruses do not cause serious symptoms so they can coexist peacefully with us for an extended period of time. In this process, they replicate without harming the host.

**Zhou:** Good. Now we have learned a lot about viruses. My next question is: what is immunity?

**Dong:** We just mentioned that our living environment is surrounded by microbes. When a person is infected by a virus, our body can effectively resist against the virus and eliminate it. This process can be sorted in two steps: first is identification; second is elimination. The identification step involves separating foreign or harmful microbes from internal microbes, and infected cells from non-infected ones. In addition, RNA viruses and DNA viruses are identified differently by our immune system. After identification, we will have immune response and immune reaction. Next, the elimination step can also be sorted into two stages: innate immunity and adaptive immunity. The innate stage is characterized by the activation of interferon production. For example, after infection with influenza we first feel body discomfort, muscle pain, lack of appetite or fever. This first response happens within the first 2–3 days. As humans, we possess an advanced second stage that is adaptive immunity. During this stage, we rely on immune cells such as lymphocytes to fight the virus.

Viruses are constantly adapting to us. In turn, we are adapting to the viruses around us.

—Chen Dong

**Zhou:** How do you evaluate a person's immune system? Are there scientific ways to quantify immune system?

**Dong:** Good question. The answer is yes. When we perform a routine physical exam, we often measure white cell count or lymphocyte count. We have determined a normal range so if your measurements fall in this range, we generally say your immune system is sound. Some viruses can compromise a person's immune system, such as HIV. In some other situations, a person's immune system can be too strong, such as with allergy.

**Zhou:** I have a question from personal curiosity. So a person's immune system can vary according to time and environment. If someone lives in a clean environment for too long, will that decrease his immune system? And conversely, if he lives in a polluted environment, will that boost his immune system?

**Shao:** Yes, our immune system needs stimulation, such as microbes in our environment. Personal hygiene is certainly important. But being a clean freak is not good for your immune system.

**Zhou:** We have discussed immunity. Next we may talk about a related issue: prevention and treatment. Experts often talk about three important aspects to prevent infectious diseases. What are they?

**Wang:** The first is identification and isolation of the patients who may release the pathogens; this step includes the early diagnosis, early treatment and early isolation like quarantine of patients and proper disposal of patients’ excrements. Second is blocking the ways of viral transmission such as holding a social distance for respiratory diseases. Third is to protect vulnerable populations from pathogen infection, including vaccinations.

**Zhou:** Good. Let's now specifically talk about COVID-19. Prof. Gao, what makes the SARS-CoV-2 virus so special compared to other corona virus?

**Gao:** This is a big topic. Since the onset of COVID-19, no scientist or public health expert predicted this virus can mutate so rapidly with so many variants. Corona viruses from the past did not mutate as fast. From its trend so far, particularly after the Omicron variant, we conjectured three scenarios to explain this. First scenario is that these variants all existed from the very beginning. Some may have been dormant. Second scenario is that some variants came from animals. Third is the viruses’ own mutation under evolutionary pressure. After people are infected, we start to develop some immunity and we start to receive vaccines. So the virus needs to adapt to our immunity in order to survive. One hypothesis regarding the source of the Omicron variant is it came from South Africa; 20% of the population in South Africa has compromised immune system due to AIDS. In a person with a normal immune system, the virus can survive for maybe three days. In an AIDS patient, the virus may survive up to a month. During such a prolonged interaction with an immune system, the virus may mutate to strange variants.

For a virus, mutation is perpetual. But in the long run, the direction is always to get weaker so it can coexist with a host.

—Yiming Shao

**Zhou:** Now we are touching on the issue of viral mutations. Does influenza mutate every year?

**Gao:** That is a good question. We encourage the public to get flu-shots every year because influenza mutates every year so we need to make different predictions every year.

**Zhou:** Does SARS-CoV-2 mutate faster than influenza?

**Gao:** Influenza is faster. But SARS-CoV-2 is getting close.

**Shao:** Regarding viral mutation, I want to add a point. For a virus, mutation is perpetual. But in the long run, the direction is always to get weaker so it can coexist with a host. A virus needs the host to survive.

**Zhou:** Another topic of great public interest is treatment. Dr. Wang, currently do we have effective drugs to treat COVID-19?

**Wang:** Yes, there are only a few antiviral drugs available for treatment of the disease. The viral infection and replication can be pathogenic at the early stage of the disease, and later the virus-mediated immune response also participates in pathogenesis. Therefore, early initiation of antiviral drugs will suppress the viral infection and prevent the disease progression. Particularly, for severely ill patients, these drugs can reduce mortality rates among them. But we do not recommend patients wait till that stage. Early treatment within the first week or two will have the best outcome.

**Zhou:** Next we talk about vaccines. Are they effective? And are they safe? Prof. Gao, would you like to start?

**Gao:** Now more than two years into the pandemic, we can let the evidence speak and let Big Data speak. The data from around the globe tell us that the safety and efficacy of the vaccines are unquestionable. There are reports of breakthrough infections or reinfections. And we know that is due to a gradual decline of antibodies after vaccination. Over time, its effect may be reduced but it is not ineffective. So we encourage people to get vaccinated.

**Zhou:** Dr. Wang, what is your experience from a clinical point of view?

**Wang:** Historically, in terms of the prevention of infectious diseases, vaccination is the most effective and most economical method. This has been proven. Specifically for COVID-19, vaccines reduce incidents of infection, severe symptoms and death. Because of breakthrough infection and reinfection by the virus, the public may have doubts about the efficacy of the vaccines. There are two reasons. First, COVID-19 is a kind of infectious disease through respiratory transmission. Even when we have developed viral-specific antibodies and immune cells after vaccination, they mainly exist inside the body and cannot exist at the respiratory tract with an effective concentration. That is why breakthrough infections and reinfections may occur. Second reason is that now we have a different way to evaluate the efficacy. In the past, we used to judge the efficacy of vaccines by looking at if people get sick after vaccination. Now we rely on tests of nucleic acid. Many positive tests came from people without any symptoms or with mild clinical symptoms. For vaccines, that is a higher bar than before.

**Zhou:** So now we have a different standard.

**Wang:** During the epidemic of COVID-19, we may count the people without clinical symptoms if their test results are positive due to extensive tests of viral nucleic acid among nearly all the population. In the past, we did not count them. That is why the vaccines may not appear as effective as we hoped. However, vaccinations are really effective. We encourage people to get vaccinated, especially for the elderly. Vaccination rate among the elderly is a shortfall. If we fill this gap, we are confident about reopening internally and outward.

**Zhou:** So the vaccines provide protection against infection and severe symptoms. Is this correct, Prof. Shao?

**Shao:** Yes. I like to say vaccines provide three levels of protection: against infection, against disease, severe symptoms and death, against transmission. When you have immunity, you have fewer viruses in your body so you release fewer to the environment, so the transmission to others is reduced.

**Zhou:** The public have questions about antibodies. Can antibodies indicate the effect of vaccines?

**Shao:** Antibody level is a good indicator for your ability against infection. Your ability against disease and severe symptoms is multifaceted, including cell immunity.

**Zhou:** After vaccination antibodies may decrease. But immune system retains memory. Is that true?

**Wang:** After vaccination, especially after the booster shot (or the third dose of vaccination), viral specific antibodies can reach a high level. Six months later, the level will naturally come down. This is a characteristic of the immune response. Without antigen stimulation, the specific antibodies will decrease over time. However, the viral-specific immunological memory can be kept *in vivo*. Once this virus reappears, the immune system will wake up and provide immune protection again.

**Zhou:** So antibody level may drop after vaccination but do not be concerned.

**Shao:** Yes. The data from Hong Kong shows that our inactivated vaccines’ efficacy against mild symptoms are lower than that of mRNA vaccines by a few dozen percentage points, but in terms of protection against severe symptoms and death, both types of vaccines are close to or better than 95%. So antibodies primarily protect against infection and our immune system provides comprehensive immune reactions casued by vaccines to fight diseases.

**Zhou:** I had the third shot more than a year ago. Time must be a factor. Do I need a fourth shot to maintain vaccine's effect? Do we need a COVID shot every year? What happens if I do not get a fourth shot?

**Shao:** Without a fourth shot, the vaccine's effect will decrease. To protect against infection and mild symptoms, it will decrease by a large amount. Against severe symptoms or death, it will decrease less. The state is conducting large scale trials regarding additional shots. Should the fourth shot be the same as before or a separate one? We are working on choosing the best vaccines and booster strategy for the public.

**Zhou:** Good. That is all for COVID-19. Now let's discuss hepatitis. Dr. Wang, you are the expert on this. So for hepatitis B and C, how are the treatments?

**Wang:** We estimate approximately100 million people in China carry the virus of hepatitis B or C. Every year we have official reports of 1 to 1.2 million patients of viral hepatitis. That makes up 1/5 of all reports of infectious diseases. In terms of treatment, hepatitis A and E can be cured completely. However, a long-term antiviral treatment is needed for the patients with chronic hepatitis B. For chronic hepatitis C, the treatment has greatly improved. It can be cured within three months with mild side effects and a reasonable cost. It is covered by our national health insurance.

For viral hepatitis B, the medication can stop its progression as long as it is taken continuously.

—Fu-Sheng Wang

**Zhou:** Do we have domestic drugs?

**Wang:** Yes. Eight years ago this treatment cost about 1.2 million Yuan by using the antiviral drugs from overseas. Now, it only costs around ten thousand, of which 70% to 80% will be covered by medical insurance. This is great news for patients with chronic hepatitis C. For chronic hepatitis B, we have good antiviral drugs to block the disease progression, preventing it from turning into liver cirrhosis or liver cancer. But the cure rate is still very low. In this field, clinicians and scientists are making progress with substantial investments from the state.

**Zhou:** So type C can be cured successfully with medication.

**Wang:** Yes. For viral hepatitis B, the medication can stop its progression as long as it is taken continuously.

**Zhou:** I heard we had a lot of children infected with type B. Do we have vaccines for it?

**Wang:** For children, the vaccine for hepatitis B works very well and is widely used. The antigen carriers are below 0.15% in China. Ninety-five percent of the vaccinated will develop a protective immune response against the viral infection. Hepatitis B virus (HBV) is a type of DNA virus. HCV is different. It is an RNA virus so it replicates more quickly. Chronic hepatitis C can be cured but there is no effective vaccine for HCV infection. A challenge for HCV infection is that around 80% of patients with chronic hepatitis C could not be diagnosed timely. If they can be diagnosed early, they can be cured in time. If not, they will have a high risk of developing liver cirrhosis or cancer.

**Zhou:** Good. Prof. Shao, now we move to AIDS. How is AIDS different from SARS-CoV-2 and hepatitis?

**Shao:** In terms of prevention and treatment, we have good vaccines for SARS-CoV-2 and hepatitis B but the antiviral drugs are not very effective. For AIDS, it is the opposite. We have excellent antiviral drugs for AIDS, but no vaccine. In fact, the first real breakthrough in all anti-virus medication was for AIDS.

**Zhou:** What is the reason?

**Shao:** I usually divide pathogens into two groups, A and B based on their ability to generate enough protective immunity or not in natural infection. SARS-CoV-2 and hepatitis B viruses (HBV) belong to group A, with characteristics of strong protective immunity, predominant asymptomatic infections and very low mortality. That is why most SARS-CoV-2 infected persons are with no or only mild symptoms and around 1% or less mortality. Dr. Wang just mentioned we have around 90 million HBV carriers, but only very few of them are actually sick. In addition, among the 700 million Chinese who experienced HBV infection (having HBV antibody), over 600 million of them have eradicated HBV. This demonstrated that our immune system is stronger than the group A pathogen in evolution. The vaccines for group A pathogens are easy to be developed through reinforcing our immune system to fight these viruses, since the evolution advantage is on our side. HIV, the virus causing AIDS, belongs to group B, to which our natural immune system is not adequate to control, with characteristics of very high symptomatic infections and mortality. Without antiviral drug treatment, more than 90% will get sick after getting HIV infection and close to 100% will die within two years after progress to AIDS. For group B pathogens, a vaccine is difficult to succeed by traditional technical means. We have to try novel design and technology to overcome the disadvantage of evolution, which is on the viral side.

**Zhou:** Are there AIDS vaccines being developed?

**Shao:** Yes. Our research team has collaborated with the largest vaccine maker in China, Sinopharm for 20 years. We have developed novel technology including immunogen design, DNA vaccine priming and live viral vector boost strategy to stimulate balanced and sustainable antibody and cell immunity to HIV. Through collaboration with Peking Union, Youan and Zhejiang University hospitals, we completed 3 phase I and 2 phase II clinical trials. Now we are preparing to apply for a phase III clinical trial.

**Zhou:** How about the cost of AIDS treatment?

**Shao:** It used to be only developed countries that had access to AIDS antiviral drug treatment due to high cost. Later, with the coordination of the World Health Organization, developing countries also gained access. Now AIDS treatment is free around the world. Our country started free AIDS treatment in 2003.

**Gao:** Free treatment sounds wonderful but the money has to come from somewhere. When the state spends it on this disease, other diseases will not have it. The total budget is fixed. So I think we still emphasize on prevention.

**Shao:** Yes, I agree. AIDS treatment has improved greatly but we need to do more. What we have now is a life-long treatment. Some people get infected in their teens. The life expectancy in China is now in the seventies so that means treatment for more than half a century. The personal cost, family cost and societal cost are too high. AIDS used to be called super-cancer. Now it is a manageable chronic disease like high blood pressure or diabetes. Patients can live a long life with continued antiviral medication. But there is drug resistance and a side effect. Before we had the antiviral medication, patients would pass away after two years of having developed into AIDS. Now they continue to live and may pass the disease to others. So now the pressure on prevention is higher than before. In that sense, the fight against AIDS is far from over. First, we need to have a vaccine based on new science and technology. That requires innovation. Second, we need to turn treatment into cure. Both efforts need international cooperation. We need to encourage more young people to engage in science and technological innovation.

**Zhou:** That is a great point. My last question is about monkey pox, which recently became a new concern of many countries. Should we worry about this? How to prevent it?

**Gao:** We should be concerned. Monkey pox was discovered in the 1970s. As of 10 October, it has spread to 107 countries and killed 26 people.

**Zhou:** How many infected?

**Gao:** Official number of the infected is 71 273.

**Zhou:** Is this death rate high or low?

**Gao:** Not high. But it can do great harm to society because transmission from skin contact is easy. That is why the WHO issued a declaration of Public Health Emergency of International Concern (PHEIC) on 23 July.

**Zhou:** Dr. Wang, how many cases do we have in China?

**Wang:** So far we have one case from abroad. But much attention will be needed for the disease.

**Zhou**: Yes. Dr. Wang, do you have any concluding remarks?

**Wang:** As a physician in infectious diseases, I often have a heavy feeling. In 1949, the life expectancy in China was 35 years old. The contribution of infectious diseases to that value was around 70%. That means when someone got infected by pathogens, death was the likely outcome. Now the life expectancy is about 77 or 78 years old. It can be attributed to successful prevention and management with scientific and medical archivements. The state has invested over 20 billion Yuan on severe infectious diseases during the last decade. This has improved the prediction, monitoring and prevention of emerging infectious diseases. Much progress has been made by the scientific community and the state.

**Zhou:** Thank you, Dr. Wang. That concludes our discussion today. I am encouraged by what I learned. Thank you very much.

## Notes

He Zhu (朱禾) is a science and news editor at the editorial office of NSR in Beijing, China.

